# Sociality influences thermoregulation and roost switching in a forest bat using ephemeral roosts

**DOI:** 10.1002/ece3.3111

**Published:** 2017-06-08

**Authors:** Danilo Russo, Luca Cistrone, Ivana Budinski, Giulia Console, Martina Della Corte, Claudia Milighetti, Ivy Di Salvo, Valentina Nardone, R. Mark Brigham, Leonardo Ancillotto

**Affiliations:** ^1^ Wildlife Research Unit Dipartimento di Agraria Università degli Studi di Napoli Federico II Portici Italy; ^2^ School of Biological Sciences University of Bristol Bristol UK; ^3^ Forestry and Conservation Cassino Italy; ^4^ Department of Genetic Research Institute for Biological Research “Siniša Stanković” University of Belgrade Belgrade Serbia; ^5^ Dipartimento di Biologia Università degli Studi di Firenze Firenze Italy; ^6^ Dipartimento di Biologia Strutturale e Funzionale Università degli Studi di Napoli Federico II Napoli Italy; ^7^ Dipartimento di Biologia e Biotecnologie “Charles Darwin” Università degli Studi di Roma “La Sapienza” Roma Italy; ^8^ Department of Biology University of Regina Regina Canada

**Keywords:** body temperature, Chiroptera, snag, torpor, tree, vespertilionids

## Abstract

In summer, many temperate bat species use daytime torpor, but breeding females do so less to avoid interferences with reproduction. In forest‐roosting bats, deep tree cavities buffer roost microclimate from abrupt temperature oscillations and facilitate thermoregulation. Forest bats also switch roosts frequently, so thermally suitable cavities may be limiting. We tested how barbastelle bats (*Barbastella barbastellus*), often roosting beneath flaking bark in snags, may thermoregulate successfully despite the unstable microclimate of their preferred cavities. We assessed thermoregulation patterns of bats roosting in trees in a beech forest of central Italy. Although all bats used torpor, females were more often normothermic. Cavities were poorly insulated, but social thermoregulation probably overcomes this problem. A model incorporating the presence of roost mates and group size explained thermoregulation patterns better than others based, respectively, on the location and structural characteristics of tree roosts and cavities, weather, or sex, reproductive or body condition. Homeothermy was recorded for all subjects, including nonreproductive females: This probably ensures availability of a warm roosting environment for nonvolant juveniles. Homeothermy may also represent a lifesaver for bats roosting beneath loose bark, very exposed to predators, because homeothermic bats may react quickly in case of emergency. We also found that barbastelle bats maintain group cohesion when switching roosts: This may accelerate roost occupation at the end of a night, quickly securing a stable microclimate in the newly occupied cavity. Overall, both thermoregulation and roost‐switching patterns were satisfactorily explained as adaptations to a structurally and thermally labile roosting environment.

## 1 INTRODUCTION

Conspecifics often share identical physiological, ecological, and behavioral requirements, so their presence (or reproductive success) provides an effective intraspecific cue for the selection of suitable habitat (Danchin, Boulinier, & Massot, 1998), including dens, roosting, or nesting sites. For social species, this might play a more important role (“social attraction hypothesis”; Danchin, Giraldeau, Valone, & Wagner, 2004) than direct habitat assessment (“public information hypothesis”; Valone, 2007). Forming conspecific groups also sets the basis for the performance of cooperative behaviors such as antipredatory vigilance and defense, group foraging, communal nursing, or social thermoregulation (Fisher, 1954; Silk, 2007).

Homeothermic species invest considerable energy in maintaining elevated, stable body temperatures, so they often use social thermoregulation, that is, group mates huddle to reduce surface‐area‐to‐volume ratio and increase the temperature of their shelter to mitigate heat loss (Hayes, Speakman, & Racey, 1992; Séguy & Perret, 2005). Along with collective nursing of young, social thermoregulation may represent the main reason for communal roosting or nesting (Kerth, Ebert, & Schmidtke, 2006; Williams et al., 2013). Heterotherms that are endotherms that exhibit reversible decreases in metabolic rate and body temperature in response to low temperatures or limited food availability (McKechnie & Mzilikazi, 2011) reduce the cost of arousals through social thermoregulation by obtaining heat from warmer group mates that began to arouse earlier (Arnold, 1993; Blumstein, Im, Nicodemus, & Zugmeyer, 2004). Heterothermy is widespread among bats as their small size and thus large surface‐area‐to‐volume ratios mean especially high energetic costs to maintain homeothermy (Altringham, 2011). Outside the period of hibernation, bats from temperate regions also employ daily torpor, that is, they exhibit daytime bouts of torpor but are active at night (Geiser, 1998).

Summer torpor might have detrimental effects on reproduction, especially on pregnant females, because embryo development may be delayed, and to a lesser extent on lactating females, because torpor might reduce milk production, yet in many temperate bat species such females still alternate between torpor and normothermic bouts (Dzal & Brigham, 2013; Rintoul & Brigham, 2014). Therefore, the energetic benefits of torpor during reproduction must outweigh its risks (reviewed in McAllan & Geiser, 2014). Thermoregulation costs add to the considerable energetic expenditure of reproduction (Gittleman & Thompson, 1988) leading to the hypothesis that social thermoregulation is especially important for pregnant and lactating females to save energy (Pretzlaff, Kerth, & Dausmann, 2010) and provide nonvolant young with a warm roosting environment (Sedgeley, 2001). Consequently, females of almost all temperate bats spend the summer communally in maternity colonies (Altringham, 2011; Kerth, 2008a).

Although colony size is often large, in tree cavities this is constrained by the limited space available, so bats that roost in trees commonly form small social subunits scattered across large forest areas (Russo et al., 2016). Tree‐dwelling bats also switch roosts frequently, to maintain social relationships (Fortuna, Popa‐Lisseanu, Ibáñez, & Bascompte, 2009; Willis & Brigham, 2004), decrease parasite loads (Reckardt & Kerth, 2007), or memorize the location of alternative roosts (Fleischmann & Kerth, 2014; Russo, Cistrone, & Jones, 2005): whatever the reason, to benefit from social thermoregulation a bat switching roosts must occupy a cavity where conspecifics are present.

**Figure 1:**
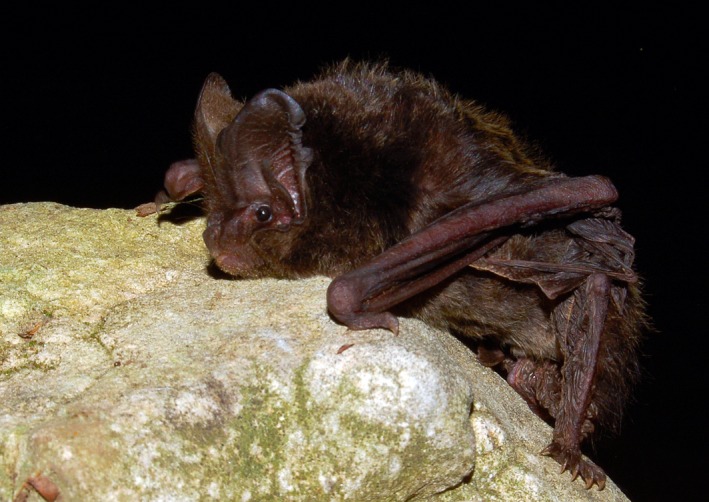
Barbastelle bat *Barbastella barbastellus*, a small‐sized vespertilionid found in Europe, Asia, and N Africa

Unlike most other bat species that roost in tree cavities formed by woodpeckers, rot, or cracks (Kalcounis‐Rüppell, Psyllakis, & Brigham, 2005), the barbastelle bat *Barbastella barbastellus* (Schreber 1774), a medium‐sized vespertilionid occurring in Europe, N Africa, and Asia (Figure 1), mostly uses spaces beneath flaking bark (Russo, Cistrone, Jones, & Mazzoleni, 2004). These cavities are ephemeral, shallow, and easily accessed by predators that rely on vision or olfaction. Bats roosting in these sites are therefore likely exposed to rain, predation, and probably cold spells, yet cavity microclimate and its relationship to ambient temperature have never been investigated.

As roosting beneath flaking bark offers little protection from rain or predators, the main advantage of this choice is that this cavity type is common in forests and subject to a faster turnover than “safer” shelters such as woodpecker holes or rot cavities (Russo et al., 2004). *Barbastella barbastellus* frequent roost switching (Russo, Cistrone, & Jones, 2007; Russo et al., 2005) supports the view that at least in forest areas where dead trees are abundant, suitable roosts are not limited (Chaverri, Quirós, Gamba‐Rios, & Kunz, 2007; Kerth & König, 1999; Lewis, 1996; Willis & Brigham, 2004). Because these cavities are shallow, however, the microclimate likely changes abruptly, making thermoregulation by roosting bats more expensive (Sedgeley, 2001). Clustering should reduce this cost by buffering the roosting environment from shifts in ambient temperature (Willis & Brigham, 2007). Social thermoregulation would therefore play an important role for bats using this roost type.

In this study, we first test the prediction that temperature beneath flaking bark will fluctuate similarly to ambient temperature, that is, that *B. barbastellus*’ preferred cavities are poorly insulated. Following Johnson and Lacki (2014), we then tested alternative hypotheses for the factors influencing thermoregulation behavior, namely that this is mainly influenced by a) the presence of roost mates and group size (hereafter called the “social hypothesis”); b) the location and structural characteristics of tree roosts (hereafter the “tree” hypothesis); c) roost cavity structure (“cavity” hypothesis); d) weather (“weather” hypothesis), or e) sex, reproductive, or body condition (“physiological” hypothesis).

Tree‐dwelling bats often exhibit fission–fusion dynamics when switching roosts (Metheny, Kalcounis‐Rueppell, Willis, Kolar, & Brigham, 2008; Popa‐Lisseanu, Bontadina, Mora, & Ibàñez, 2008), meaning that at least some roost mates maintain group cohesion and move together to a new site (Kerth, 2008b). The decision, adopted through “unanimous” or “majority” rules, could be signaled by swarming near roosts after foraging (Naďo & Kaňuch, 2015). Maintaining social cohesion would facilitate search, signaling, and occupation of new cavities and perform cooperative behaviors including those that might prove vital in an ephemeral roosting environment such as information transfer. Coordination among socially related bats might accelerate roost occupation at the end of a night, when roosts are coldest, quickly producing a stable microclimate in the newly occupied cavity and increasing survival probability of any nonvolant young (Kunz & Lumsden, 2003). We therefore predict that social cohesion will prevail in roost‐switching *B. barbastellus* during the reproductive season as an adaptation to a structurally and thermally labile roosting environment.

## 2 MATERIALS AND METHODS

### 2.1 Study area

The study was carried out at the Abruzzo, Lazio, and Molise National Park (41°47′20″N, 13°46′33″E), Italy, in a mountainous area of the central Apennines of ca. 700 ha dominated by a *Fagus sylvatica* old forest where previous studies of *B. barbastellus* have taken place (Russo, Cistrone, Garonna, & Jones, 2010; Russo et al., 2004, 2005, 2007, 2015). Other tree species besides beech, for example sycamores (*Acer pseudoplatanus*), are uncommon. Most forest in the study area has not been managed since 1956 or is subject to only limited and selective logging. Other habitats in the study area comprise forested pasture, that is, pastures associated with old trees and shrubs, and open forest, where trees were historically pruned traditionally by “shredding.” Further details on the study area are given in Russo et al. (2004, 2005, 2015).

### 2.2 Capture and tagging

Bats were captured in 2.5 × 6 and 2.5 × 12 m mist nets set at dusk for 2–6 hr near cattle troughs frequently used by bats as drinking sites (Russo et al., 2004). For each captured bat, we measured body mass and forearm length, respectively, with a digital scale to the nearest 0.1 g and a caliper to the nearest 0.1 mm. Reproductive status was ascertained following Racey (1988): males were categorized either as reproductive or as nonreproductive, while females were classified, respectively, as pregnant, lactating, postlactating, and nonreproductive.

Bats were tagged with temperature‐sensitive (LB2XT, LB2NT, and LB2T, Holohil Systems Inc., Carp, Canada) radio tags attached with Torbot (Cranston, Rhode Island, USA) surgical cement between the shoulder blades after partly trimming the fur; tag mass was between 0.33 and 0.43 g, falling within 5% of a bat's body mass (Aldridge & Brigham, 1988; O'Mara, Wikelski, & Dechmann, 2014). Subjects were released within ca 10 min after tagging. Bat capture and processing were authorized by the Italian Ministry for the Environment and the Protection of Land and Sea and the Park's direction.

### 2.3 Location of roosts, measurement of roost characteristics, and emergence counts

Bats were tracked on foot during the daytime to find roosts using a three‐element Yagi antenna connected to a Sika receiver (Biotrack Ltd., Wareham, UK). Once a roost tree was found, its location was recorded using a GPS, and the exact roost position was assessed based on signal strength and direction. In most cases, we observed bats inside the cavity or leaving it at emergence time. At each roost tree, following Russo et al. (2004) we recorded elevation, canopy closure (visually assessed at the base of the tree and recorded as percent closure), trunk diameter at breast height (DBH), and roost aspect (expressed in degrees as the angle between the north direction and that of the middle point of cavity entrance). For cavities whose location was unambiguously identified, we also recorded type (rot cavity, crack, or space beneath loose bark) and height above ground. At most roosts, we assessed group size from recordings of evening emergence taken with a night‐shot function Sony PC 115 digital video camera (Russo et al., 2007).

### 2.4 Thermal profiling

Tagged bats were continuously monitored for 2–10 days (mean ± *SD*: 4.1 ± 2.5 days) for each roost they used (i.e., every time a bat switched roost, at least two thermal profiling days were undertaken at the new location). The pulse emission rates of tags changed according to the subject's skin temperature (*T*_skin_), which was assessed using unit‐specific calibration curves provided by the manufacturer. We also verified the reliability of calibration in the laboratory for a subset of tags (Stawski & Geiser, 2012). We timed the duration of 21 pulses three times every 15 min for all bats from dawn to dusk emergence and calculated hourly means of such measurements, resulting in 15–17 values per bat/tracking day (Otto, Becker, & Encarnação, 2013; Nardone et al., 2015). At the same time intervals, ambient temperature (*T*_a_) was measured with a digital thermometer (precision: 0.1°C) placed in the shade near the roost at ca. 1.5 m above ground. We could not measure roost internal temperatures (*T*_roost_) because most cavities were located too high making them difficult to access. However, we extrapolated a relationship between the outer surface temperature of flaking bark and that of the space beneath it so that the latter could be inferred from the former. We did this for 30 cavities 2–4 m above ground of the type used by *B. barbastellus*, including some that had been used as roosts based on radiotracking. Every hour we measured internal cavity temperature with a 0.1°C precision digital probe thermometer positioned inside the cavity, taking care that thermal sensor did not touch roost internal surface; at the same time, we took a thermal image of the outer surface of the cavity with a FLIR T240 thermal camera (FLIR Systems, USA) mounted on a 1.5‐m tripod at ca. 3 m from the base of the tree. Outer temperatures were extracted from digital images with FLIR Research IR software. We then fitted a power regression model including outer (independent variable) and internal (dependent variable) temperatures, respectively (see Figure S1 in Supporting Information). We used this relationship to infer *T*_roost_ from thermal images of roost cavities for which no direct measurements of internal temperature could be taken. We took one thermal image per hour of all roosts used by bats whose *T*_skin_ we were measuring.

### 2.5 Relationship between ambient and roost temperatures

To evaluate the hypothesis that cavity temperature in spaces beneath flaking bark fluctuates with external temperature, we explored the relationship between hourly *T*_a_ and *T*_roost_ with a Pearson correlation test and compared them with a Student's *t* test for paired observations. Roost insulation was expressed as the daily mean difference between *T*_roost_ and *T*_a_.

### 2.6 Testing potential thermoregulation drivers

We applied the equation proposed by Willis (2007) to assess the temperature of torpor onset (*T*_onset_) for each bat on each day. We used this value to obtain: a) occurrence of torpor, that is, a binary value indicating whether a bat entered torpor (1, present; 0, absent) on a given day, b) number of torpor bouts per day, c) total daily time spent torpid, d) torpor depth, that is, the difference between *T*_onset_ and the minimum *T*_skin_ reached on a given day. Following Johnson and Lacki (2014), each response was tested separately as the dependent variable in five different generalized linear mixed‐effect models (GLMMs), each representing one of the competing a priori hypotheses we formulated for thermoregulation behavior. In all models, roost and individual bat identities were included as random effects. The five hypotheses were then ranked in order of decreasing parsimony using Akaike's information criterion adjusted for small sample sizes (AICc), and Akaike differences (Δi) and weights (wi) as ranking parameters (Burnham & Anderson, 2002). To avoid zero inflation of models, analyses concerning responses b–d included only days when bats did use torpor.

The alternative hypotheses we formulated were tested as follows. Social hypothesis: we used a binary variable describing roosting condition, that is, whether a bat was roosting alone or in group, and numbers of bats in the group as a factor nested within the former factor; tree hypothesis: variables comprised site elevation, canopy closure, DBH, tree height, and exposition; cavity hypothesis: variables comprised roost type, roost internal mean daily temperature, roost height, and roost insulation; weather hypothesis: variables comprised minimum and mean daily temperatures and precipitation (binary classified as presence or absence of rain during a monitoring day); physiological hypothesis: variables comprised sex, reproductive status (reproductive vs. nonreproductive/postreproductive), and body condition expressed as a scaled mass index (Peig & Green, 2009). To evaluate variable importance within each model, we checked parameter estimates, errors and *p* values from the GLMMs outputs, considering all variables scoring *p* < .05 as significant.

### 2.7 Roost and social fidelity

We tested whether the association among tracked bats arose from the independent decision of individuals to select favorable roosts (passive association, or roost fidelity) or from group decisions made among group members (active association, or social fidelity). We employed a dataset of 71 roost‐switching events recorded from 102 individuals tagged between 2001 and 2016 in the study area, including those recorded by Russo et al. (2005, 2007) and those observed during field work carried out for the present study.

As bats never reused the same roost in a given year (pers. obs.), roost fidelity was calculated by modifying the formula of Chaverri and Kunz (2006): FID = ((2*STAY) − (1*MOVE))/(STAY+MOVE), where STAY is the number of times a bat was observed in the same roost on consecutive days, and MOVE is the number of times an individual moved to a previously unidentified roost.

Males roost solitarily during summer (Russo et al., 2005) so we restricted our analyses to females, specifically to those which shared roosts with at least another tagged bat and that switched roost at least once during a tracking session (3–24 days). Social fidelity was measured as the degree of cohesive movement of pairs of individuals roosting together following Campbell, Akbar, Adnan, and Kunz (2006). For each dyad of tagged bats, we selected a focal subject as the bat observed over more consecutive days and calculated social fidelity as the ratio between the number of times that a dyad of tagged bats was found roosting together on two consecutive tracking days and the number of times the focal subject switched roost. For each dyad, we considered the reproductive status of individuals as well as whether these differed or not between the two members. We explored the occurrence of differences in roost and social fidelity using one‐way ANOVAs. For roost fidelity, reproductive status and sex were entered as explaining variables, while for social fidelity we used dyad type (featuring two conditions, i.e., same vs. different reproductive statuses), status combination in the dyad (comprising all combinations of the two females’ reproductive status, pregnant, lactating, postlactating or nonreproductive), and season (classified as early or late reproductive season following Willis & Brigham, 2004) as factors. Differences among status combinations were tested with Bonferroni post hoc tests for multiple comparisons. We also used Pearson's correlation to assess whether social fidelity was correlated with the number of switching events. Significance for all tests was set at *p* < .05. In all cases, mean values are given ±1 standard deviation.

## 3 RESULTS

### 3.1 Roosts used by *B. barbastellus*

We captured and tagged 17 adult *B. barbastellus* in July/August 2016, comprised of five males, two pregnant, two nonreproductive, and eight lactating females. Bats were monitored over 3–24 consecutive days (mean ± *SD*: 8.76 ± 6.15 days). We found 78 roost trees at a mean altitude of 1,492 ± 122.0 m a.s.l. (range 1,262–1,697 m a.s.l.). We ascertained the cavity used by bats for 71 (91%) trees. Bats always roosted in beech trees, mostly beneath flaking bark (*n* = 56), and more rarely in crevices (*n* = 14) or rot cavities (*n* = 1). Roost cavities were 7.4 ± 3.9 m (range 1.7–17.3 m) above the ground.

### 3.2 Relationship between ambient and cavity temperatures

**Figure 2:**
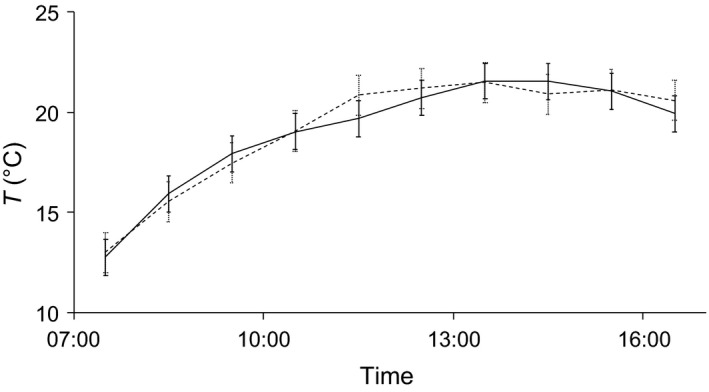
Mean hourly ambient (solid line) and internal roost (dashed line) temperatures of *Barbastella barbastellus* tree roosts. Temperatures were measured while bats were roosting. Error bars represent 1 standard deviation. Differences between roost and ambient temperatures are not significant (paired Student's *t* test, n.s.)

Cavities beneath exfoliating bark were poorly insulated and strongly affected by ambient temperatures based on the small values of daily *T*_roost_ − *T*_a_ (0.8 ± 2.7°C, range 4.5–4.8°C). Hourly *T*_roost_ was positively correlated with *T*_a_ (Pearson's *r* = .90, *p* < .001) and did not differ significantly from it (*t* = 0.59, n.s.). *T*_roost_ ranged between 9.0 and 25.6°C and reflected values of *T*_a_ (range 8.0–30.1°C) over time (Figure 2).

### 3.3 Testing potential thermoregulation drivers

We simultaneously measured *T*_skin_, *T*_roost_, and *T*_a_ for 17 bats over 70 bat days. Bats made substantial use of torpor, which was recorded for 58.8% of monitoring days by bats of both sexes and all reproductive classes. The presence and number of conspecifics represented the most likely driver of thermoregulation patterns (Table 1): the social hypothesis model best explained all responses except the number of torpor bouts, which was still explained (delta AIC value <4) but less effectively (second rank) than by the physiological hypothesis model (first rank). The poorest performing model was that associated with the cavity hypothesis, which only explained torpor depth. Intermediate levels of support were received by the physiological hypothesis, which best explained the numbers of torpor bouts recorded and also predicted torpor duration, and by the climate hypothesis, which explained torpor depth and duration (Table 1).

**Table 1:** Akaike's information criterion (AICc) scores, differences (Δ_*i*_), weights (*w*_*i*_), and number of parameters (*K*) from five linear mixed models describing four different thermoregulatory responses of *Barbastella barbastellus*. **: best performing model; *: valid model (Δ < 4)

Thermoregulatory response	Hypothesis	*K*	AICc	Δ_*i*_	*w*_*i*_
Torpor use	Social	2	218.9**	0.0	0.971
	Physiological	3	220.1*	1.2	0.015
	Cavity	4	223.4	4.5	0.009
	Weather	2	234.4	15.5	0.004
	Tree	5	250.1	31.2	0.001
Numbers of torpor bouts	Social	2	179.7*	1.1	0.213
	Physiological	3	178.6**	0.0	0.556
	Cavity	4	189.9	11.3	0.026
	Weather	2	189.5	10.9	0.097
	Tree	5	200.3	21.7	0.008
Torpor depth	Social	2	315.7**	0.0	0.732
	Physiological	3	333.6	15.9	0.100
	Cavity	4	319.0*	3.3	0.055
	Weather	2	317.7*	2.0	0.111
	Tree	5	361.4	45.7	0.002
Torpor duration	Social	2	391.1**	0.0	0.881
	Physiological	3	396.6	5.5	0.088
	Cavity	4	415.4	24.3	0.015
	Weather	2	392.9*	1.8	0.010
	Tree	5	408.4	17.3	0.006

Use and patterns of torpor were influenced by several variables (Table 2). Namely, bats roosting in groups used torpor on fewer days than those roosting alone (37.5 vs. 82.4% of tracking days, respectively, *p* < .01). Bats in groups also used more torpor bouts per day (1.5 ± 0.7 vs. 1.0 ± 0.8, *p* < .05) as well as shallower (2.1 ± 2.6 vs. 5.2 ± 2.5°C; *p* < .001) and shorter torpor bouts than bats roosting alone (3.9 ± 3.3 vs. 6.8 ± 4.5 hr/day, *p* < .05). Finally, bats in larger groups used shallower torpor than those in smaller groups (*p* < .05).

**Table 2:** Results from generalized linear mixed models testing the effects of social, roosting tree, cavity, weather, and physiological characteristics upon four thermoregulatory responses of roosting *Barbastella barbastellus*. *p < .05; **p < .01; ***p < .001; n.s.** = **not significant

Model	Hypothesis	Variable	Estimate	Error	*Z*	*p*
Torpor use	Social	Condition	−0.33	0.12	9.01	**
		Colony size	−0.01	0.02	0.01	n.s.
	Tree	Elevation	0.03	0.00	5.97	*
		Canopy closure	0.01	0.02	0.41	n.s.
		DBH	0.03	0.07	0.02	n.s.
		Exposition	0.01	0.01	0.13	n.s.
		Tree height	0.02	0.01	1.18	n.s.
	Cavity	Roost type	−0.02	0.19	3.96	n.s.
		Internal temperature	−0.03	0.03	1.57	n.s.
		Roost height	−0.01	0.02	0.01	n.s.
		Insulation	−0.03	0.05	0.01	n.s.
	Weather	Min temperature	−0.02	0.02	2.90	n.s.
		Precipitation	0.25	0.18	1.49	n.s.
	Physiological	Sex	0.27	0.19	7.32	**
		Reproductive status	−0.20	0.16	0.93	n.s.
		Body condition	0.05	0.05	0.86	n.s.
Numbers of torpor bouts	Social	Condition	−0.35	0.11	3.09	*
		Colony size	−0.00	0.04	0.02	n.s.
	Tree	Elevation	−0.01	0.00	5.38	*
		Canopy closure	0.00	0.00	0.09	n.s.
		DBH	0.05	0.11	0.03	n.s.
		Exposition	0.00	0.00	0.05	n.s.
		Tree height	0.04	0.02	3.44	n.s.
	Cavity	Roost type	−0.10	0.50	1.28	n.s.
		Internal temperature	−0.05	0.05	1.09	n.s.
		Roost height	0.01	0.08	0.26	n.s.
		Insulation	0.02	0.08	1.12	n.s.
	Weather	Min temperature	−0.02	0.03	2.29	n.s.
		Precipitation	0.16	0.28	1.49	n.s.
	Physiological	Sex	0.39	0.30	2.80	n.s.
		Reproductive status	−0.12	0.27	0.07	n.s.
		Body condition	0.11	0.08	1.55	n.s.
Torpor depth	Social	Condition	−1.43	1.36	15.48	***
		Colony size	−0.26	0.14	6.08	*
	Tree	Elevation	−0.36	0.35	1.09	n.s.
		Canopy closure	0.49	0.13	0.03	n.s.
		DBH	0.40	0.49	0.15	n.s.
		Exposition	−0.43	0.48	1.48	n.s.
		Tree height	0.42	0.67	0.00	n.s.
	Cavity	Roost type	−2.10	0.92	1.60	n.s.
		Internal temperature	−0.23	0.13	10.67	**
		Roost height	−0.15	0.08	2.36	n.s.
		Insulation	−0.59	0.23	1.15	n.s.
	Weather	Min temperature	−0.11	0.10	6.20	*
		Precipitation	0.99	0.89	0.80	n.s.
Physiological	Sex	2.76	0.91	10.33	**
	Reproductive status	−0.55	0.80	0.41	n.s.
	Body condition	0.45	0.26	2.84	n.s.
Torpor duration	Social	Condition	−1.66	3.48	4.74	*
		Colony size	−0.64	0.33	1.46	n.s
	Tree	Elevation	0.03	0.00	4.47	*
		Canopy closure	0.02	0.02	1.07	n.s.
		DBH	0.78	0.74	0.49	n.s.
		Exposition	−0.00	0.00	3.64	*
		Tree height	0.05	0.11	0.02	n.s.
	Cavity	Roost type	−3.05	4.50	0.04	n.s.
		Internal temperature	−0.39	0.30	6.81	*
		Roost height	−0.18	0.17	2.36	n.s.
		Insulation	−0.67	0.60	0.08	n.s.
	Weather	Min temperature	−0.35	0.17	11.96	**
		Precipitation	0.52	1.46	0.05	n.s.
	Physiological	Sex	3.68	2.88	1.00	n.s.
		Reproductive status	0.01	2.70	0.01	n.s.
		Body condition	0.67	0.71	0.77	n.s.

**Figure 3:**
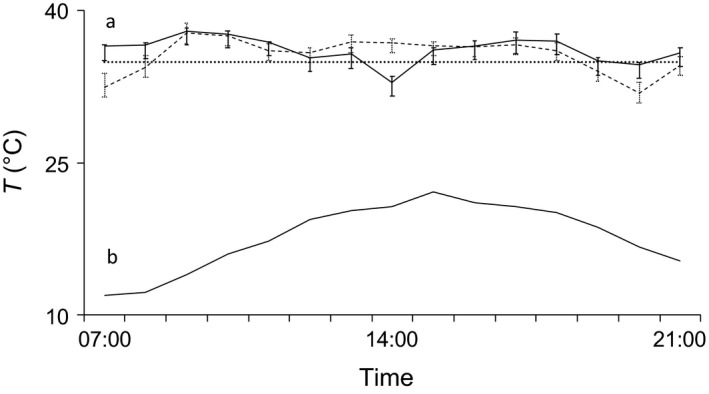
Simultaneous daily thermal patterns of two female *Barbastella barbastellus* roosting together in the same social group. Solid line in the upper part of the figure (a) shows skin temperature of a nonreproductive female, dashed line that of a lactating female; point line: torpor onset threshold. Solid line in the lower part of the figure (b) shows ambient temperature. Error bars show ±1 standard deviation. Sunrise and sunset times on sampling day were, respectively, 05.45 and 20.36

**Figure 4:**
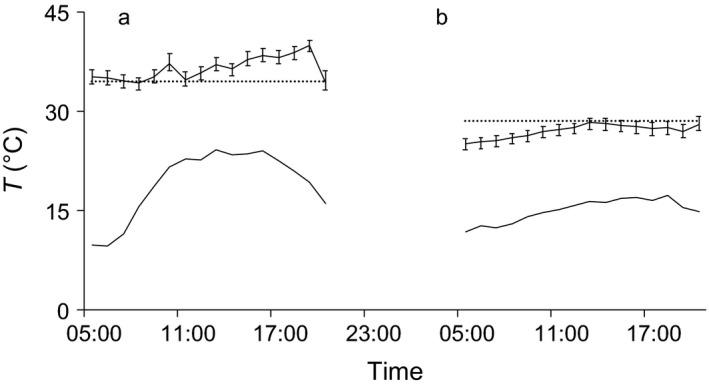
Patterns of skin temperature (solid lines with error bars) of the same female *Barbastella barbastellus* over two consecutive days, that is, when roosting in a group of six bats (a) and alone (b); ambient temperature (solid lines with no error bars in the lower part of the figure) and torpor onset threshold (dotted line) are also represented. Skin temperatures were not measured at night (between 21.00 and 04.00), when bats were active, but only in daytime, when they were roosting. Error bars show ±1 standard deviation

Nonreproductive females (*n* = 2) always associated with lactating females, and females in both conditions showed overlapping thermoregulatory patterns over 7 days of monitoring (Figure 3). In August, following a heavy rain, two postlactating females left their colonies to roost alone for one and 2 days, respectively, during which time they used torpor extensively (Figure 4). They then re‐joined groups that probably comprised the same former roost mates judging from the presence of other tagged group mates and retuned the same thermoregulatory pattern as before, mostly remaining homeothermic.

**Figure 5:**
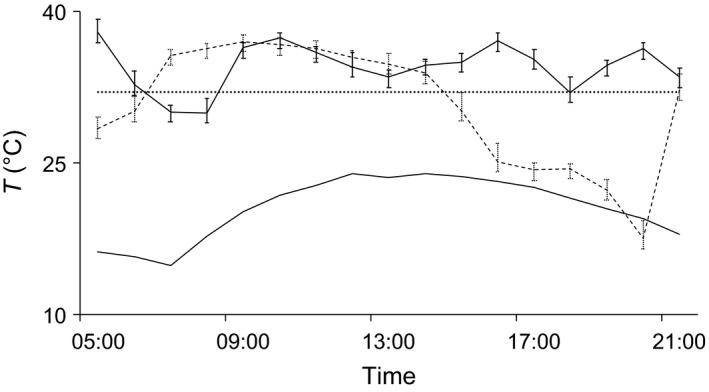
Simultaneous daily patterns of skin temperature of a male and female *Barbastella barbastellus* roosting in two different roosts. The upper part of the graph shows skin temperatures of male (dashed line) and female (solid line with error bars) and the torpor onset threshold (dotted line). The solid line with no error bars represents ambient temperature (Ta). Error bars show ±1 standard deviation. Times of sunrise and sunset on sampling day were, respectively, 05.48 and 20.34

Torpor in males was significantly (*p* < .01) more frequent (80.0% vs. 40.5% of tracking days,) and deeper (5.0 ± 2.8 vs. 2.6 ± 1.9°C) than in females (Figure 5). Torpor was also longer (*p* < .01) and deeper (*p* < .05) at lower minimum ambient temperatures. Of the roost characteristics we considered, only roost internal temperature influenced torpor, which was longer (*p* < .05) and deeper (*p* < .01) in colder roosts. Bats roosting in trees at higher elevations showed longer (*p* < .05) but less frequent (*p* < .05) torpor bouts.

### 3.4 Roost and social fidelity

Roost fidelity differed significantly between the sexes (*F*_1,44_ = 4.18, *p* < .05): males (*n* = 11) were less faithful to roosts (0.23 ± 0.33) than females (*n* = 42; mean roost fidelity: 0.46 ± 0.25) and switched roosts more often (*F*_1,44_ = 6.11, *p* < .05), using the same tree for 2.2 ± 1.8 (range 1–7 days) consecutive days versus 3.1 ± 4.5 days recorded for females (range 1–17 days). Roost fidelity of females was independent of reproductive condition (*F*_3,44_ = 1.06, n.s.).

**Figure 6:**
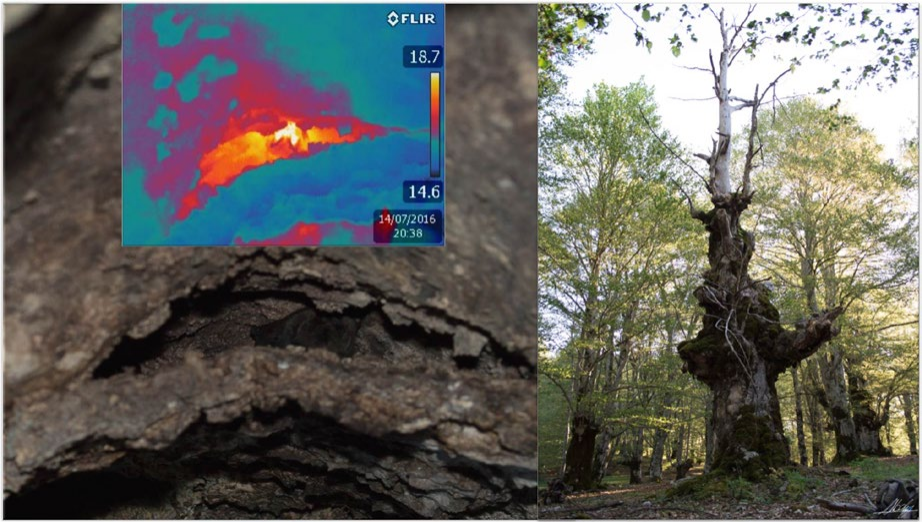
A male *Barbastella barbastellus* (left) roosting solitarily beneath the exfoliating bark of a beech tree (right). The bat is also visible in the thermographic image of the cavity (upper left box)

**Figure 7:**
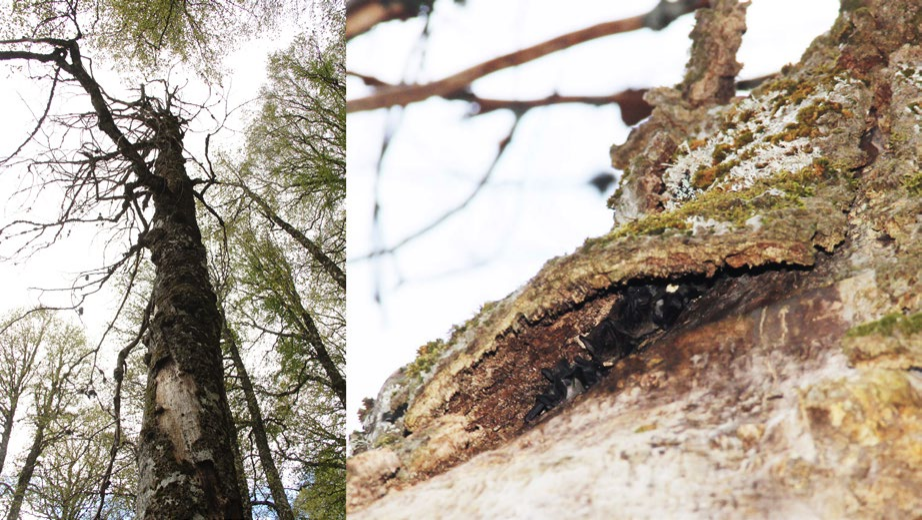
Snag (left) and flaking bark cavity (right) used by a group of female *Barbastella barbastellus* and their pups; at least three pups are visible

We analyzed the strength of association between 33 female dyads that switched roosts one to four times. Unlike males, that typically roosted alone, all females roosted in groups, including nonreproductive individuals (*n* = 7), except the two postlactating individuals tracked in 2016 mentioned above, which roosted alone for one and 2 days, respectively (Figures 6, 7). Females sharing the same groups exhibited a high degree of association (0.82 ± 0.28; range 0.33–1.00) that did not depend on dyad type (*F*_1,29_ = 4.49, n.s.). However, the combination of reproductive conditions in the dyad did have an effect on fidelity (*F*_1,29_ = 5.39, *p* < .05; Table 3): Significant differences were found between dyads comprised of at least one postlactating female and all other categories (all *p* < .05). The strength of association between females was not correlated with the number of roost switches (Pearson's *r* = .35, n.s.) and decreased in late lactation or postlactation (*F*_1,29_ = 173.91, *p* < .001).

**Table 3:** Values of social fidelity index for 33 dyads of female *Barbastella barbastellus* expressed as mean ± standard deviation. Numbers in parentheses indicate sample size for each category; n.o.** = **not observed, that is, combination not present in the sample

	Pregnant	Lactating	Postlactating	Nonreproductive
Pregnant	1.00 ± 0.00 (1)	–	n.o.	n.o.
Lactating	0.90 ± 0.22 (4)	1.00 ± 0.00 (15)	0.40 ± 0.10 (8)	–
Postlactating	n.o.	–	0.40 ± 0.10 (2)	n.o.
Nonreproductive	n.o.	0.90 ± 0.20 (3)	n.o.	n.o.

## 4 DISCUSSION

### 4.1 Summer torpor and homeothermy in *B. barbastellus*

We found that *B. barbastellus* alternate between torpor and homeothermy while roosting in summer. Despite its potential interference with embryo development and or lactation (McAllan & Geiser, 2014), torpor is employed by pregnant or lactating females of many temperate species (Audet & Fenton, 1988; Chruszcz & Barclay, 2002; Lausen & Barclay, 2003; Willis, Brigham, & Geiser, 2006; Willis, Voss, & Brigham, 2006; Dzal & Brigham, 2013; Klug & Barclay, 2013; Johnson & Lacki, 2014) including *B. barbastellus*. Patterns of skin temperature were best modeled based on the presence and number of conspecifics. Reproductive females roosting in small groups exhibited shorter and shallower torpor bouts than those recorded for males and nonreproductive females that roosted alone, while torpor patterns of communally roosting nonreproductive or postreproductive females were identical to those of reproductive females, counter to our expectations based on individual physiology and energetic requirements. We conclude that, as for *Eptesicus fuscus* (Willis & Brigham, 2007), for reproductive groups of *B. barbastellus* social thermoregulation represents the main factor shaping thermoregulatory patterns.

Willis and Brigham (2007) found no differences between maximum and minimum *T*_roost_, spatial variability in this factor, or in predicted energy expenditure between more versus less preferred tree cavities used by *Eptesicus fuscus*. However, cavity temperature increased by as much as 7°C when bats were present relative to unoccupied cavities corresponding to savings in thermoregulation up to ca. 53% of the daily energy budget. In agreement with previous work (Russo et al., 2004, 2010), in this study *B. barbastellus* mostly roosted beneath flaking bark. Although we could not test whether the presence of bats buffered roost microclimate, this is highly probable because, as we showed, cavities are poorly insulated. In this environment, huddling may be crucial for mitigating the energetic costs of homeothermy or to accelerate arousal from torpor (e.g., Chruszcz & Barclay, 2002; Solick & Barclay, 2006). We therefore argue that social thermoregulation for *B. barbastellus* might be even more important than for species using well‐insulated cavities, which buffer roost temperatures relative to ambient (Willis & Brigham, 2007).

Torpor depth was dependent on group size and was shallower for larger groups as expected because these led to greater energy savings (Willis & Brigham, 2007). Forming numerous aggregations would therefore maximize benefits, as predicted by the “group augmentation” hypothesis (Kokko, Johnstone, & Clutton‐Brock, 2001), albeit group size in *B. barbastellus* is strongly constrained by the narrow space available beneath exfoliating bark and is typically <30 bats (Russo et al., 2005).

Pregnant females face high costs of homeothermy as they cannot use prolonged torpor without delaying embryo development, so they take advantage of communal roosting (e.g., Webber et al., 2016; Willis, Brigham, et al., 2006; Willis, Voss, et al., 2006). Interference between torpor and individual physiological status appears more acceptable for lactating females, which may therefore exhibit longer and deeper torpor bouts than do pregnant females (Dzal & Brigham, 2013). Our sample size of pregnant females was too small to assess such differences, and since those we tagged were in late pregnancy, they probably began lactating soon after we started to monitor them. Noticeably, lactating females still made considerable use of homeothermy despite its high energy costs, so they surely benefit from social thermoregulation. Persistence of homeothermy in lactating females is classically explained as a way to avoid torpor‐induced reduction in milk production (McAllan & Geiser, 2014; Racey & Swift, 1981; Wilde, Knight, & Racey, 1999), but here we propose two additional, nonmutually exclusive explanations. First, advantages may be indirect, mostly concerning nonvolant juveniles, which would attain a more rapid growth and a larger body size by exploiting the warm roosting microclimate generated by normothermic adults (Ransome, 1998; Lausen & Barclay 2006; Russo & Ancillotto, 2015). Lactation might also explain the occurrence of more frequent torpor bouts in groups, probably because lactating females arouse (and interrupt torpor) more frequently to suckle or groom the young. In our case, the number of torpor bouts per day was best explained by the physiological model, which incorporated individual reproductive status.

Evolutionary pressure exerted by predators on bats selecting what appears to be an unsafe roosting environment might also help explain homeothermy in lactating bats and especially in post‐ or nonreproductive females roosting communally. Although torpor is associated with reduced predation risk as it is normally performed in secluded areas or safe shelters, out of the reach of predators (Turbill, Bieber, & Ruf, 2011), this situation is occasionally reversed when predators specialize on gaining access to torpid individuals which, being mostly incapable of moving, are easy prey. For instance, badgers excavate torpid ground squirrels *Spermophilus richardsoni* (Michener, 2004) and great tits (*Parus major*) kill and eat hibernating pipistrelle bats in caves (Estók, Zsebők, & Siemers, 2009).

*Barbastella barbastellus* roosting beneath exfoliating bark are often very exposed, occasionally almost protruding out of their roosts (pers. obs.), so they may be easily detected by predators relying on vision or olfaction such as martens or snakes. Homeothermic subjects remain reactive so they may quickly fly to escape, as was occasionally recorded in response to an approaching observer (Russo et al., 2004). The antipredatory value of being homeothermic in unsafe shelters, that is, under potentially high predation risk should therefore not be dismissed.

Russo et al. (2004) found that roost selection by *B. barbastellus* depends on tree condition (dead beech trees were preferred) and height (roost trees were taller than random trees), while cavity selection relies on cavity type (those under exfoliating bark were preferred), height (cavities at higher heights above ground were preferred), and entrance direction (cavities facing south were preferred). Taller trees, as well as cavities at greater height above ground, may keep bats safer from predators besides offering a warm microclimate through greater exposure to solar radiation, the same reason that might make bats prefer southern‐facing cavities (Russo et al., 2004). The preference for snags is clearly linked with the frequent presence of exfoliating bark in such trees. It is important to note that while roost selection analyses such as that of Russo et al. (2004) compare structures of used versus available cavities, highlighting why bats neglect certain cavities among those potentially available, the present work explains which aspects of the roosting environment play an important role in influencing summer torpor.

### 4.2 Group cohesion and roost‐switching behavior

Roost ephemerality as well as the presence of other species using the same roost type might explain frequent roost switching in *B. barbastellus* (Russo et al., 2005, 2007); for instance, *Myotis sodalis* using bark roosts move more often than those roosting in more stable structures such as crevices (Kurta & Murray, 2002). By switching roosts frequently, bats might reinforce their memory of where roosts used previously are located, check their current conditions, or locate new suitable cavities (Russo et al., 2005). We found that females are more loyal to roosts than solitary males, perhaps because most females we tracked were lactating and at this stage roost switching is reduced to avoid moving nonvolant young which probably increases predation risk (Russo et al., 2005, 2007).

Thermoregulatory benefits gained through communal roosting are so crucial that they may have exerted major influences on evolution of sociality in these mammals (Kerth, 2008a). It is therefore legitimate to argue that social thermoregulation has likely influenced fission–fusion dynamics. Our analysis confirms that, as proposed in previous radiotracking studies on *B. barbastellus* (Russo et al., 2005), cohesion is often maintained despite roost switching. While this reduces the chances of interacting with a larger network of conspecifics (e.g., Fortuna et al., 2009; Kerth & Konig, 1999; Rhodes, 2007; Willis & Brigham, 2004), it secures availability of a comfortable, energetically convenient roosting environment and increases the likelihood—or perhaps speeds up the process—of gathering a group at a new roosting site. Swarming behavior, needed to advertise the location of the new roost (Naďo & Kaňuch, 2015), also occurs in *B. barbastellus* (Russo et al., 2005). Of course, other social factors may have influenced the onset of social cohesion in roost‐switching *B. barbastellus*, including other forms of cooperative behavior such as antipredatory (Lind & Cresswell, 2005) or communal foraging (Dechmann, Kranstauber, Gibbs, & Wikelski, 2010) and nursing (Wilkinson, 1992) strategies.

### 4.3 Future prospects

*Barbastella barbastellus* is an ideal species to analyze the effect of roosting environment on sociality. We undertook this study in an area where one of the most important Italian populations occurs, most probably because forest management has specifically targeted snags favoring their presence. We observed thermoregulatory and roost‐switching patterns that may be explained as responses to structurally and thermally labile roosts, which bats still prefer probably because they are so abundant to outweigh such disadvantages; on the other hand, the latter may be mitigated through sociality. Such cavities are often present in standing dead trees that must be easy to locate, reducing the amount of energy needed to find suitable roosting sites (Russo et al., 2005, 2007). *Barbastella barbastellus* may sometimes roost in different habitats, such as managed forest (Russo et al., 2010) or even clay badlands (Ancillotto, Allegrini, Serangeli, Jones, & Russo, 2015; Ancillotto, Cistrone, et al., 2015), where studies analogous to ours should be undertaken for comparison.

Kinship among group members (Kerth, 2008b; Rossiter, Jones, Ransome, & Barratt, 2002) or persistence of cryptic social subunits established among adults or at an early life stage (Ancillotto, Serangeli, & Russo, 2012; Ancillotto, Allegrini, et al., 2015; Ancillotto, Cistrone, et al., 2015) might play an important role in influencing social interactions and maintaining cohesion and should also be addressed in future work. We highlight that the importance of thoroughly understanding roosting behavior trespasses its physiological and eco‐ethological interests because many forest bat species are threatened by forestry (Russo et al., 2016): Sustainable management may only be achieved improving comprehension of how and why bats select essential resources in forest ecosystems.

## AUTHOR CONTRIBUTIONS

DR, LC, VN, and LA conceived the experiment; DR, LC, CM, GC, MDC, IDS, IB, and LA collected the data; LA, VN, GC, and CM performed the statistical analyses; DR, RMB, and LA wrote the manuscript.

## CONFLICT OF INTEREST

None declared.
