# Supplementary material for: Sociality influences thermoregulation and roost switching in a forest bat using ephemeral roosts
Source: Ecol Evol. 2017 Jun 8;7(14):5310–21. doi: 10.1002/ece3.3111 (PMC5528228; doi:10.1002/ece3.3111)
Supplement: Supplementary file 1 [file ECE3-7-5310-s001.docx]

**Supplementary material**

**
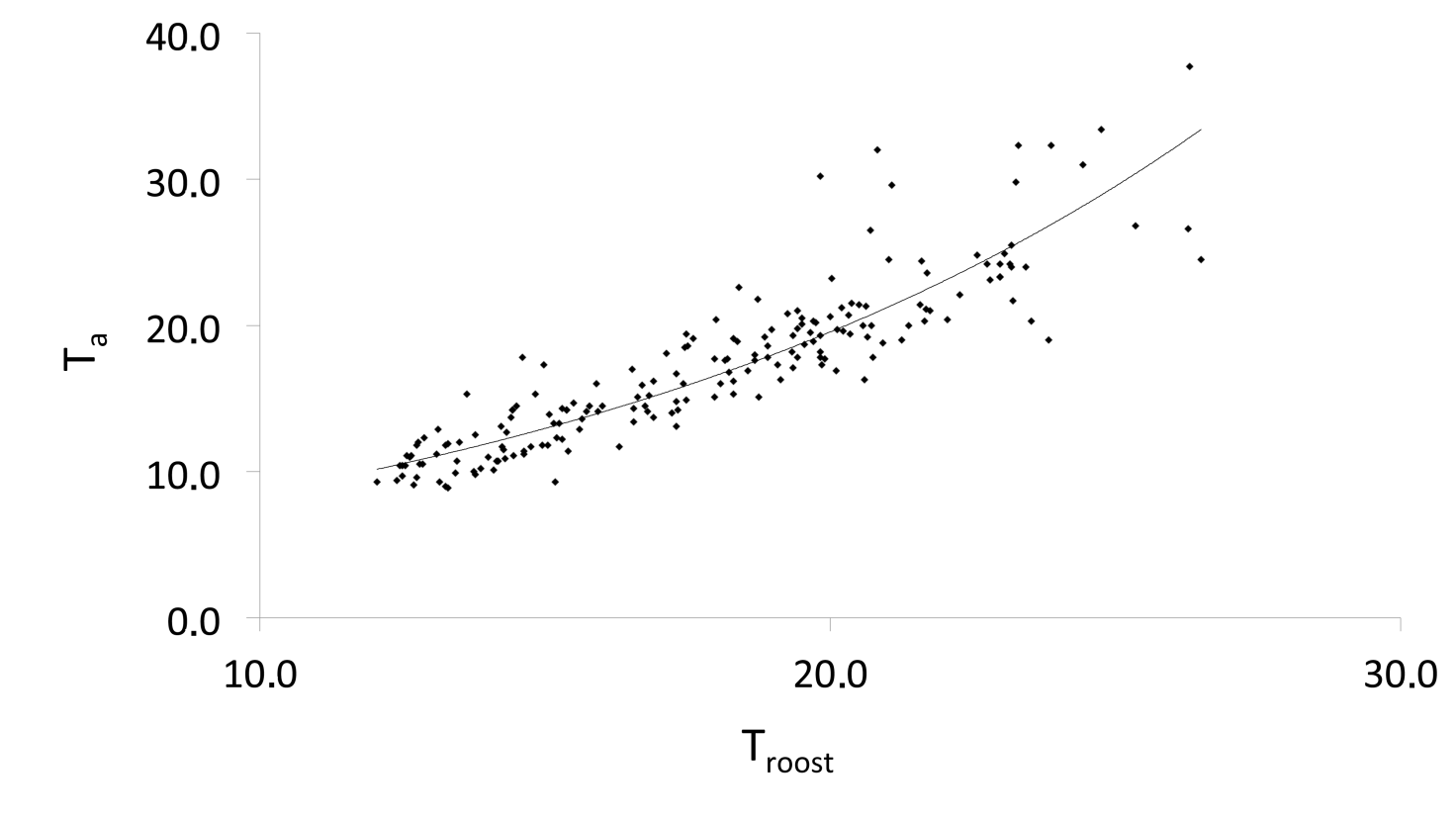
**

**Figure S1.** Relationship between hourly ambient temperature and temperature recorded in the space beneath loose bark (n=30) of *Fagus sylvatica* trees. The trend-line of the best performing model (power regression: y=3.77e^0.0824x^, R^2^=0.85) is shown. The best model was selected from among linear, exponential, logarithmic, power and polynomial (2^nd^ and 3^rd^ degree) alternatives according to the greatest R^2^ value.
